# An unusual case of bilateral anterior uveitis related to moxifloxacin: the first report in Latin America

**DOI:** 10.3205/oc000069

**Published:** 2017-07-07

**Authors:** Carlos M. Rangel, M. Margarita Parra, Gabriel Frederick, Alejandro Tello, Clara L. Varón

**Affiliations:** 1 Retina and Vitreous Department, Fundación Oftalmológica de Santander Clínica Carlos Ardila Lulle, Bucaramanga, Colombia; 2 Fundación Oftalmológica de Santander Clínica Carlos Ardila Lulle, Bucaramanga, Colombia; 3 Anterior Segment and Refractive Surgery Department, Fundación Oftalmológica de Santander Clínica Carlos Ardila Lulle, Bucaramanga, Colombia

**Keywords:** uveitis, quinolones, adverse effect, pigment dispersion syndrome

## Abstract

**Objective:** To report a case of bilateral anterior uveitis secondary to oral moxifloxacin.

**Methods:** Case report.

**Results:** A 54-year-old female presented bilateral anterior uveitis following a 10-day course of oral moxifloxacin. She developed a bilateral anterior uveitis associated with pigment dispersion syndrome and iris transillumination.

**Conclusions:** Drug-induced uveitis is one of the causes of anterior uveitis. Uveitis related to fluoroquinolones is a rare entity, there are few cases reported in the literature, this is the first case reported in Latin America.

## Introduction

The term uveitis describes a group of inflammatory disorders; anterior uveitis is the most common form of intraocular inflammation and the nongranulomatous subtype is the most prevalent. Several causes have been described, most of these are related to autoimmune disorders and infections [1]. In recent years, other causes of uveitis have been reported and there has been growing interest in the study of drug-induced uveitis. Cidofovir, bisphosphonates, ribabutin, sulfonamides, and oral fluoroquinolones are the most studied as triggering medications for its development [2]. The association of fluroquinolones and uveitis has been described since 2004 [3]; the mechanism underlying drug-induced uveitis is generally unclear, although mechanisms related to phototoxicity and autoimmune predisposition are proposed [4]. A syndrome of bilateral iris transillumination and iris depigmentation has been identified with the use of fluoroquinolones [4]. The recognition of drug-induced uveitis will lead the clinician to select the best treatment for each case and stop the causative agent. An accurate and detailed medication history should be obtained in all patients with uveitis.

The following is a case description of a female patient who developed anterior uveitis related to oral moxifloxacin

## Case description

A 54-year-old woman underwent a mandibular bone graft and dental implant placement, and was prescribed 800 mg oral moxifloxacin once a day. Ten days later she presented intense bilateral eye pain, photophobia and red eye. She self-medicated with eye drops (neomycin sulfate 3,500 IU/ml, polymyxin B sulfate 6,000 U/ml, dexamethasone 0.1%) with partial improvement. One month later, she went to the emergency room at Fundación Oftalmológica de Santander – FOSCAL (Floridablanca, Colombia) referring persistence of symptoms. Her corrected distance visual acuity (CDVA) was 20/20 in both eyes. Slit-lamp examination demonstrated ciliary injection, clear cornea with some descemet membrane folds, endothelial pigment, clumps of pigment and white blood cells in the anterior chamber, and posterior synechiae. Gonioscopy showed an important amount of pigment in trabecular meshwork. Intraocular pressure was 12 mmHg in both eyes and dilated fundus examination was normal. She received atropine 1% three times a day and prednisolone 1% every 2 hours for five days, which was gradually tapered. The results of infectious and immunological markers were negative, except for an increased erythrocyte sedimentation rate (Table 1 (Tab. 1)).

One month later, ocular inflammation resolved and symptoms disappeared.

**Figure 1:**
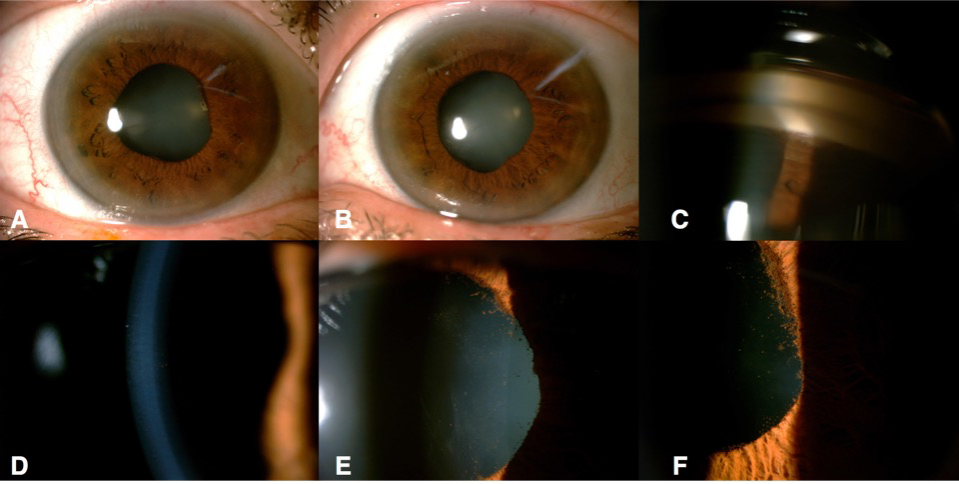
Clinical photography of both eyes A. Right eye shows mild conjunctival injection with dyscoria secondary to posterior synechiae. B. Left eye shows no conjunctival injection with dyscoria secondary to posterior synechiae. C. Gonioscopy reveals presence of pigment in the trabecular meshwork. D. High-magnification photo shows retroqueratic pigment. E. High-magnification photo shows pigment over lens anterior capsule. F. High-magnification photo shows wide posterior synechiae.

The patient was evaluated one year later. Her DCVA was 20/20 both eyes, the presence of pigment in the trabecular meshwork and endothelium persisted, and iris transillumination was still present (Figure 1 (Fig. 1)). No recurrences during follow-up were register. 

## Discussion

Moxifloxacin is a broad-spectrum antibiotic classified within the group of fluoroquinolones, its mechanism of action is the inhibition of the activity of DNA gyrase and DNA topoisomerase IV, necessary to bacteria DNA replication [5]. There are not many reports in the literature about the association of moxifloxacin and induced uveitis. This association was first described in 2004 by Bringas et al., who reported a clinical case of acute bilateral uveitis in a 77-year-old female patient after receiving treatment for a neumococic pneumonia with oral moxifloxacin [3]. It has been found more prevalent in women than in men, with a mean age of 54 years, usually developing in a period of 0 to 20 days after the start of treatment [6]. The more frequently described clinical features of this condition are the diffuse iris transillumination, variable amounts of pigment dispersion, and mid-dilated atonic pupils [6]. In our patient, these findings were evident since the first evaluation and they were persistent at one year of follow-up. Whereby we can suggest that in cases of bilateral anterior uveitis in patients with overall normal results of diagnostic tests, and without history of autoimmune or infectious diseases, preponderance should be given to an accurate drug history. Still, we must keep in mind that bilateral anterior uveitis may be the first presentation of autoimmune diseases, and when the initial workup is done these results may be negative to all autoimmune diseases, but afterwards some exams can become positive. Moxifloxacin-induced uveitis can also present with elevated intraocular pressure in up to 50% of patients, presumably related to pigment dispersion [7]. Our patient did not present ocular hypertension. 

The etiology of moxifloxacin induced uveitis is unclear. Autoinmune predisposition, phototoxicity, and/or viral infection are amongst the proposed mechanisms. Classically uveitis is associated with an inflammatory/autoimmune or infectious origin. However, it is important to keep in mind the association of some medications with uveitis, to make an appropriate approach in these uncommon cases, before considering it as idiopathic.

## Conclusions

Bilateral anterior uveitis is an adverse rare side effect of systemic fluoroquinolone administration. This is a rare entity, with a few cases published and according to our knowledge the patient presented corresponds to the first case reported in Latin America.

## Notes

### Competing interests

The authors declare that they have no competing interests.

## Figures and Tables

**Table 1:**
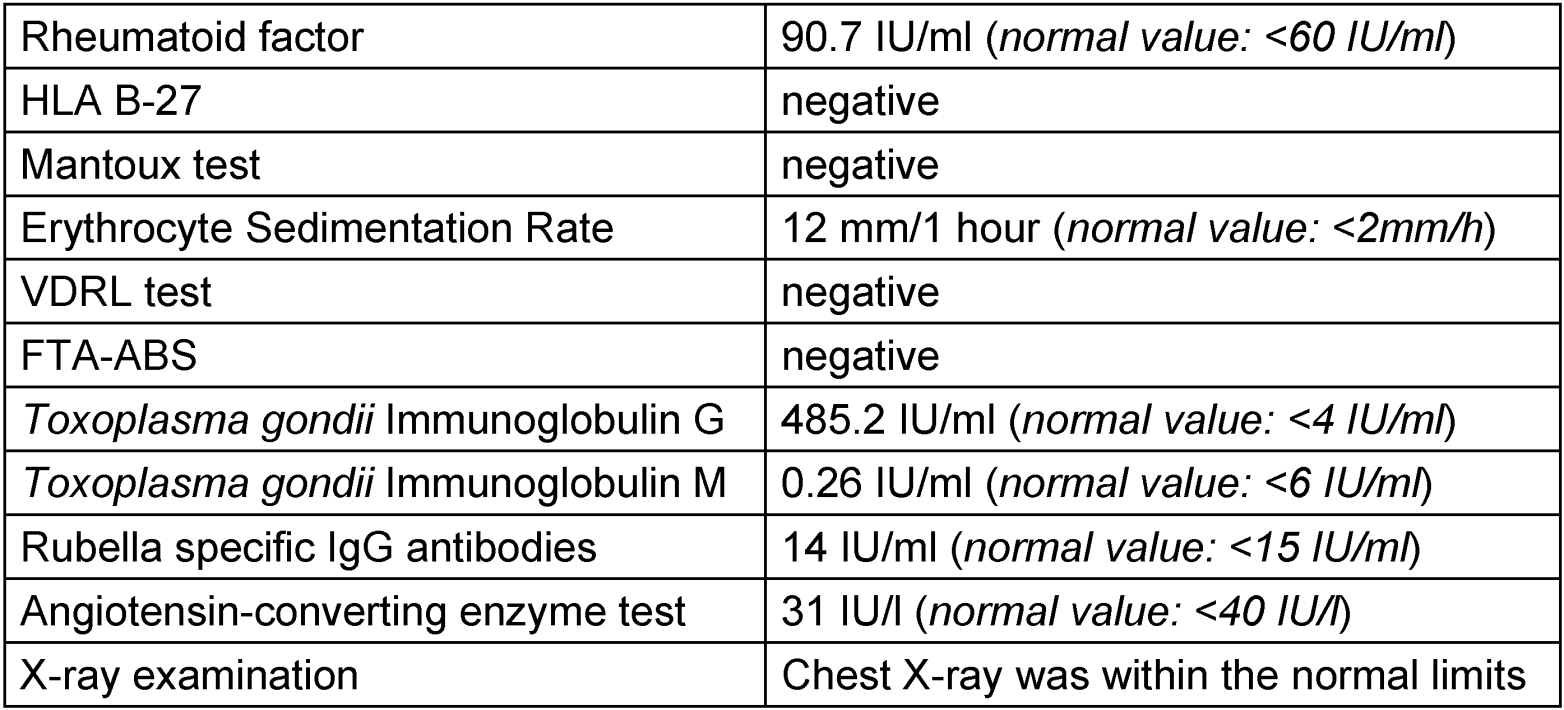
Infectious and immunological markers
